# Detection of Diphtheritic Polyneuropathy by Acute Flaccid Paralysis Surveillance, India

**DOI:** 10.3201/eid1909.130117

**Published:** 2013-09

**Authors:** Farrah J. Mateen, Sunil Bahl, Ajay Khera, Roland W. Sutter

**Affiliations:** Johns Hopkins University, Baltimore, Maryland, USA (F.J. Mateen);; World Health Organization, Geneva, Switzerland (F.J. Mateen, R.W. Sutter);; World Health Organization National Polio Surveillance Project, New Delhi, India (S. Bahl);; Ministry of Health and Family Welfare, New Delhi (A. Khera)

**Keywords:** diphtheria, Corynebacterium diphtheriae, bacteria, paralysis, diphtheritic polyneuropathy, neurology, Guillain-Barré syndrome, immunization, acute flaccid paralysis surveillance system, India

## Abstract

Diphtheritic polyneuropathy is a vaccine-preventable illness caused by exotoxin-producing strains of *Corynebacterium diphtheriae*. We present a retrospective convenience case series of 15 children (6 girls) <15 years of age (mean age 5.2 years, case-fatality rate 53%, and 1 additional case-patient who was ventilator dependent at the time of last follow-up; median follow-up period 60 days) with signs and symptoms suggestive of diphtheritic polyneuropathy. All cases were identified through national acute flaccid paralysis surveillance, which was designed to detect poliomyelitis in India during 2002–2008. We also report data on detection of diphtheritic polyneuropathy compared with other causes of acute flaccid paralysis identified by this surveillance system.

Diphtheria is caused by toxin-producing strains of the bacterium *Corynebacterium diphtheriae* and is spread by human-to-human contact (respiratory secretions and cutaneous lesions). Before the advent of vaccination with diphtheria toxoid in the 1940s, ≈1 in 20 persons in temperate zones had diphtheria in their lifetime and 5%–10% of cases led to death (*1*). The last case of diphtheria in the United States was in 2003 in a traveler who returned from Haiti (*2*). However, diphtheria remains a health concern among immigrants, travelers, and those with incomplete immunity and vaccination coverage in many regions. Adults may also be increasingly at risk for diphtheria because of waning immunity or incomplete immunization, especially in outbreak situations (*3*).

Although diphtheria is preventable by vaccination, only 24% of countries worldwide reached the targeted >80% routine coverage of all districts for diphtheria-tetanus-pertussis (DTP3) vaccine in 2011 (*4*). In 2004, the World Health Organization (WHO) reported 5,000 deaths caused by diphtheria, all of which were in children <5 years of age (*5*). However, reporting of diphtheria is variable, and some countries report cases inconsistently because of limited recognition among health care workers and no dedicated surveillance systems (*5*). It is likely that many cases are not reported.

Diphtheria is clinically considered to be a biphasic illness with initial symptoms of low-grade fever, sore throat, neck swelling, nasal twang, and usually ipsilateral palatal paralysis. The time between the first symptoms of diphtheria and the onset of polyneuropathy is deemed the latency period. Diphtheritic polyneuropathy occurs in ≈20% of patients with diphtheria. It is considered more likely with higher release of exotoxin (*6*,*7*) The classic features of diphtheritic polyneuropathy include sensory and motor signs and symptoms, most notably acute flaccid paralysis (AFP) with reduced or absent deep tendon reflexes. Limb paralysis from a segmental demyelinating process occurs with temporal regularity, with onset and resolution 35–140 days after the onset of bulbar signs and symptoms (*6*,*7*). Although serious, diphtheritic polyneuropathy is not consistently fatal and may resolve. Death from diphtheria occurs by parasympathetic dysfunction of the vagal nerve with cardiac arrhythmias, myocarditis, or from respiratory paralysis caused by laryngeal involvement (*2*).

We report a case series of diphtheritic polyneuropathy in children in India identified by routine screening for AFP, which was performed to achieve eradication of poliomyelitis in India. The clinical characteristics of diphtheritic polyneuropathy are presented in detail to remind clinicians of the key diagnostic features of this major cause of neuropathy and the value of a throat examination in persons with AFP.

Cases of diphtheria with associated flaccid paralysis are reported to the polio eradication program in India as part of routine surveillance of AFP in children ≤15 years of age. All children with new-onset weakness in ≥1 limbs or facial weakness are reported by a network of health facilities that have been established in all districts of India for AFP reporting. Cases are reviewed for poliomyelitis; this review includes results of fecal testing for poliovirus serotyping. Case-patients, or primary providers for young or severely ill children, are interviewed. All case-patients with AFP are examined in detail by district medical officers trained by government/WHO surveillance officers.

On the basis of results of fecal testing, preliminary clinical findings, and course of the illness, a final diagnosis is established by the district government/WHO medical officers in tandem with local physicians. For patients from whom fecal samples are not collected in time to diagnose poliomyelitis (cases with inadequate samples or inadequate signs and symptoms), the final diagnosis is made by an expert review committee of neurologists, virologists, pediatricians, and epidemiologists convened by the WHO–India National Polio Surveillance Unit in New Delhi, India (*8*,*9*). This committee comprises a minimum of 3 senior experts, usually professors of major academic centers in India, to determine the final diagnosis of children with AFP and inadequate fecal specimens. The committee uses no specific algorithm or diagnostic criteria for every case but comes to a consensus on the most likely diagnosis on the basis of clinical, laboratory, and epidemiologic data.

All cases in this series were given a final clinical diagnosis of diphtheritic neuropathy. The European Union case definition (2002) for the National Diphtheria Surveillance category of probable diphtheria was used to look for diphtheria: “a clinically compatible case that is not laboratory confirmed and does not have an epidemiological link to a laboratory case.” Diphtheria is clinically defined “as an upper respiratory tract illness characterized by sore throat, low grade fever, and an adherent membrane of the tonsils, pharynx or nose or non-respiratory diphtheria; cutaneous, conjunctival, otic, and genital lesions” (*10*). The cases reported represent a convenience sample of cases suggestive of diphtheritic polyneuropathy during 2002–2008 in which Guillain-Barré syndrome or poliomyelitis was first suspected. For many case-patients, details of antecedent sore throat, adherent membrane, and respiratory and systemic features were not available because the investigation began only after reporting of AFP and surveillance dedication to poliomyelitis. All cases were diagnosed as diphtheria clinically by at least the treating physicians and the expert review committee in New Delhi.

The cases reported were included because of chart accessibility at the National Polio Surveillance Unit and accessibility of paper files from large storage containers in off-site warehouses. All cases were classified as cases having inadequate fecal specimens for poliovirus testing.

Clinical features were extracted from information obtained by medical officers at the time of clinical presentation. Preselected clinical history, physical examination, baseline demographics, and laboratory findings were obtained from standardized reporting forms. All surviving case-patients were evaluated and caregivers were interviewed at a follow-up visit 60 days after diagnosis per usual protocols during AFP surveillance in India. Death information was also ascertained at 60 days. A neurologist (F.J.M.) extracted all relevant and available clinical information from the medical and surveillance records and retrospectively assigned a Guillain-Barré syndrome disability outcome score (*11*).

Fifteen children with features suggestive of diphtheritic paralysis were identified (6 girls, average age 5.2 years, age range 2.3–14.5 years) in 9 states and union territories of India (Table 1, Appendix). The proportion of AFP cases with inadequate signs or symptoms believed to represent diphtheria compared with other causes detected in a single year is shown in the Figure.

**Table 1:** Clinical characteristics of 15 children with diphtheritic polyneuropathy, India, 2007–2011*

Patient	Age, y/sex	Paralysis description (worst motor power)	Tones/reflexes	Fever at onset	Neck swelling	Other symptoms of diphtheria	CSF/NCS	Respiratory involvement	GBS disability score	Outcome at 60-d follow-up (no. days death occurred postparalysis onset)
1	5/F	Symmetric limb weakness (NA)	NA	N	N	Throat pain, nasal regurgitation, nasal intonation, and twang with speech	NA	Y	6	Initially improved to independent sitting, standing, and holding head; then died from presumed respiratory failure (46)
2	3/M	Hypotonic, areflexic, symmetric weakness of all limbs (MRC 2)	↓/↓	Y	N	Neck flop, prior sore throat, nasal regurgitation, inability to speak	NA	Y	5	Ventilator dependent
3	4/M	Descending, asymmetric lower extremity paralysis (MRC 4)	↓/NL	N	Y	Nasal voice, nasal regurgitation of food, progressive weakness, and inability to walk after throat symptoms improved	NA	Y	6	Died from respiratory failure (15)
4	3/M	Symmetric, hypotonic, areflexic paralysis of the extremities (MRC 3)	↓/↓	Y	Y	Nasal voice and regurgitation with feeding ≈15–20 d after neck swelling	NA	N	3	No clinical improvement, remained hyporeflexic and hypotonic in limbs
5	4/M	Symmetric, hypotonic, diffuse weakness, unconscious (unable to test)	↓/↓	Y	Y	Hyponasal speech, difficulty swallowing, nasal regurgitation 20 d after neck swelling	NA	Y	6	Died from cardiorespiratory failure while ventilator dependent (36)
6	6/M	Symmetric, hypotonic weakness, lower extremity weakness (MRC 3)	↓/↓	Y	Y	Neck swelling and fever for 13 d, flaccid paralysis developed 30 d later with persistent voice change	NL/NA	Y	6	Died from cardiorespiratory failure while ventilator dependent (18)
7	6/F	Symmetric, descending, hypotonic, lower extremity weakness (MRC 4)	↓/NL	Y	Y	Nasal regurgitation and speech twang with enlarged glands and cervical adenopathy; weakness 13 d later	NL/NL	Y	6	Died from unclear reasons, presumed cardiorespiratory failure (37)
8	6/F	Symmetric, ascending, hypotonic weakness (MRC 3)	↓/↓	Y	Y	Nasal twang, swallowing difficulty for 1 mo, then weakness involving legs and hands for <1 wk	NA	N	3	Strength improved by 1 point on MRC scale in upper and lower extremities
9	6/M	No limb weakness (MRC 5)	NL/NL	Y	Y	Fever for 2 d, then sudden voice change with nasal regurgitation for 2–3 d, before pain and swelling of neck 2 weeks earlier	NA	N	0	Persistent palatal palsy
10	2/F	Symmetric, descending, hypotonic weakness (MRC 4)	↓/↓	Y	N	Fever followed by nasal regurgitation and difficulty swallowing, progressive weakness in all limbs developed 2 d later	NA	N	4	Able to stand but requires support to walk
11	4/M	Symmetric, hypotonic, lower worse than upper extremity weakness with prominent sensory symptoms (MRC 3 and 4)	↓/↓	N	Y	Throat and bulbar symptoms preceding limb weakness; throat swab negative	NL/demyelinating	Y	6	Serum given without noticeable improvement; died from cardiorespiratory arrest (13)
12	4/M	Descending, symmetric, lower extremity predominant weakness (MRC 4)	↓/↓	N	Y	Nasal regurgitation preceding weakness	NA/mixed axonal and demyelinating	Y	6	Died from respiratory failure (21)
13	5/F	Symmetric, diffuse, hypotonic weakness (MRC 4)	↓/↓	Y	Y	Nasal regurgitation	NL/NA	Y	6	Died from presumed cardiorespiratory failure (6)
14	14/M	Complete flaccidity, areflexia, atonia; EMG showed no spontaneous motor activity or recruitment of motor unit potentials (MRC 0)	↓/↓	Y	N	History of positive throat swab result for *Corynebacterium diphtheria*	NL/demyelinating	Y	5	No spontaneous muscle activity; no response to IVIg
15	2/F	Symmetric, 4-limb, lower extremity, predominant weakness (MRC 3 in lower extremities)	NL/↓	N	N	Nasal regurgitation, speech change, and difficulty swallowing with fever, paralysis in <7 d	NL/demyelinating	N	4	Unable to walk

*CSF, cerebrospinal fluid; NCS, nerve conduction study; GBS, Guillain-Barré syndrome; NA, not available; N, no; Y, yes; MRC, Medical Research Council scale score; ↓, decreased or absent deep tendon reflexes; NL, normal; EMG, electromyogram; IVIg, intravenous immunoglobulin.

**Figure:**
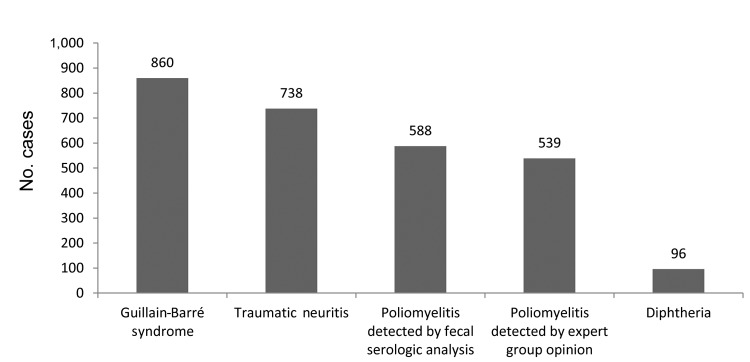
Reported cases of acute flaccid paralysis in children <15 years of age in India caused by selected factors affecting the peripheral nerve and anterior horn cell, taken from discarded cases in which fecal samples were inadequate to confirm or refute poliomyelitis on the basis of timing of samples or other reasons, 2008. Cases indicated as diphtheria were deemed diphtheritic polyneuropathy by the Expert Review Committee and were suggestive of diphtheritic polyneuropathy but may not meet standard case definitions such as those derived in the European Union. Values above bars are numbers of cases.

The average time from paralysis onset to maximal weakness was 10 days (SD 9 days, range 1–30 days). Clinical features included fever (60%, n = 9) and laryngeal, pharyngeal, or nasal symptoms (100%, n = 15). Data on exudative pseudomembranes in the pharynx were incomplete at the time of evaluation of AFP and were not part of the screening examination in poliomyelitis surveillance. There were no cases in which cutaneous diphtheria was noted before the onset of paralysis.

Paralysis was ascending (33%, n = 5), descending (27%, n = 4), or uncertain (40%, n = 6). It was most often symmetric (93%, n = 14). The number of paralytic limbs involved was 4 in 73% (n = 11), 2 in 20% (n = 3), and 0 in 13% of cases (n = 2, i.e., palatal and pharyngeal paralysis only). The mean lowest motor power was 3.3/5 in the lower extremities (range 0–5) and 3.7/5 in the upper extremities (range 0–5). Most case-patients also had hyporeflexia or areflexia (73%, n = 11) and hypotonia of the arms (60%, n = 9) and legs (80%, n = 12).

All case-patients had been vaccinated with oral poliovirus vaccine; these case-patients had a mean of 16 doses before AFP onset (range 4–40 doses). No patients had poliovirus identified in fecal samples. Cerebrospinal fluid was analyzed for 6 patients, and results were within reference ranges for all patients (mean ± SD protein level 32 ± 12.8 mg/dL, range 16–49 mg/dL; mean ± SD glucose level 71 ± 17 mg/dL, range 40–89 mg/dL; mean ± SD leukocyte count 2 ± 2 cells/mL, range 0–5 cells/mL). Nerve conduction studies were performed for 6 patients. Results were categorized by the examining physician in India as demyelinating (n = 4), mixed demyelinating and axonal (n = 1), and normal (n = 1) at the time of the study.

Cardiorespiratory failure occurred in 10 (67%) patients and was the attributed cause of death for 8 (53%) patients; an additional patient was ventilator dependent at the time of last follow-up evaluation. Mean ± SD time to death was 24 ± 14 days from AFP onset (range 6–46 days). Guillian-Barré syndrome disability scores among survivors showed persistent disability without major improvement in most patients (median disability score 4 [bedridden or chair bound], median follow-up 60 days, mean 54 days).

## Diphtheria in India

During 2007–2011, a total of 55 countries worldwide, including 10 high-income countries, reported >20,000 cases of diphtheria to WHO (*4*). India reported the most cases of diphtheria of any country (n = 17,926) and more than all of the other highest reporting countries combined (Table 2). During 1998–2008, India had 19%–84% of the global incidence of diphtheria. Since the Expanded Program on Immunization was introduced in the late 1970s, the number of diphtheria cases in India has decreased but diphtheria remains a major cause of illness and death. There were 39,231 reported cases in 1980, 8,425 in 1990, 5,125 in 2000, and 3,485 in 2011 (*12*).

**Table 2:** Countries reporting >100 cases of diphtheria, 2007–2011*

Rank in no. reported cases	Country	World Bank gross national income per capita level (2010)	World Health Organization region	No. reported cases
1	India	Low	Southeast Asian	17,926
2	Indonesia	Lower middle	Western Pacific	1829
3	Nepal	Low	Southeast Asian	710
4	Iran	Upper middle	Eastern Mediterranean	380
5	The Philippines	Lower middle	Western Pacific	329
6	Sudan	Low	African	243
7	Bangladesh	Low	Southeast Asian	190
8	Russia	Upper middle	European	169
9	Ukraine	Upper middle	European	167
10	Haiti	Low	Americas	151
11	Pakistan	Low	Eastern Mediterranean	136
12	Brazil	Upper middle	Americas	127
13	Thailand	Lower middle	Southeast Asian	105
14	Afghanistan	Low	Southeast Asian	104

*Source: World Health Organization Immunization Assessment, Surveillance, and Monitoring: Diphtheria (http://www.who.int/immunization_monitoring/data/data_subject/en/index.html).

Most persons with diphtheria in India are either not vaccinated or have only partial immunization from DTP or DTP combination vaccines (*13*). In this case series, vaccination status for DTP was not unreported during routine AFP surveillance because the programmatic focus was on poliomyelitis. In India in 2011, >4 million children did not receive a first dose of DTP vaccination and >2.5 million did not receive a third dose (*12*,*14*). Although this number represents the minority of all children in India, this country had the highest number of children worldwide who had not received DTP vaccine (*12*,*14*).

## Clinical Aspects of Diphtheritic Polyneuropathy

Diphtheritic polyneuropathy is a toxic complication of initial infection with *C. diphtheriae* resulting from hematogenous dissemination of intracellular toxin subunit A. Transport down the axon of newly synthesized protein with eventual destruction of the myelin sheath accounts for the delay in neuropathic symptoms (mean 8 weeks) after initial infection (*15*). Diphtheritic polyneuropathy is potentially reversible, and surviving patients often report few to no neurologic symptoms (*16*). Our report attempts to underscore the key clinical features of diphtheritic polyneuropathy, including initial sore throat; nasal, laryngeal, and pharyngeal involvement; neck swelling with the classic bull neck appearance; and the potentially long time course between initial infection and neuropathic symptoms.

Diphtheritic polyneuropathy is generally considered a demyelinating neuropathy with proximal to distal spread of weakness and prominent sensory features. However, in children and in resource-limited settings, simply examining for pharyngeal exudate in the setting of AFP may help lead to a clinical diagnosis, throat swab, and appropriate management with antidiphtheroid serum and antimicrobial drugs when available (*17*,*18*). Isolation of the bacterium from a throat swab specimen is unlikely in most cases. Other reports of diphtheritic neuropathy in India have been bacteriologically confirmed for 15%–39% of patients (*19*–*21*). Indiscriminate use of antimicrobial drugs before the diagnosis of diphtheria may make culture positivity even more difficult (*17*,*19*) and is also possible in this case series because many parents in India first seek the advice of local healers and health care workers before seeking formal medical evaluation.

In the United States, diphtheria antitoxin is available only through the Centers for Disease Control and Prevention and should be given as soon as possible after diagnosis of diphtheria or suspected diphtheria (*2*,*18*). Although rapid administration of diphtheria antitoxin reduces the case-fatality rate for respiratory diphtheria, antitoxin administration after day 1 of diphtheritic polyneuropathy shows no benefit (*22*). Many countries no longer store diphtheria antitoxin for therapeutic use. A recent survey including 47 countries in which the diphtheria antitoxin stock was known during 2007–2008 found that only 57% stocked antitoxin, including countries in which diphtheria antitoxin is still produced (*23*). India produces and exports diphtheria antitoxin, but local patients are often unable to pay for this therapy (*20*,*23*).

## Detection of Diphtheritic Neuropathy by AFP Surveillance Systems

Our study demonstrates that diphtheritic neuropathy can be detected through existing AFP surveillance systems designed to detect poliomyelitis and may be pragmatically expanded to include diphtheritic neuropathy in children <15 years of age. Several additional features make diphtheritic polyneuropathy more difficult to detect than poliomyelitis. Awareness of diphtheritic polyneuropathy may be lower than that of poliomyelitis among health care workers and surveillance officers. Laboratory testing for diphtheria requires special growth media and is less sensitive and less specific for diagnosis of diphtheria than fecal testing for poliomyelitis, especially for patients who have received antimicrobial drugs. In the case of diphtheritic polyneuropathy, nasopharyngeal involvement precedes development of neuropathy and may be resolved by the time AFP develops. Isolated cranial nerve involvement is notable in patients with diphtheritic polyneuropathy and is not currently a major focus of AFP surveillance. Case definitions were developed in higher-income settings and may not be developed for or by low-income practitioners for general use in resource-constrained settings.

Nonetheless, simple additions to the current screening for AFP in diphtheria-endemic areas, including dedicated questions about DTP or DTP combination vaccination status, contacts with known diphtheria case-patients, cutaneous lesions, and nasopharyngeal symptoms might inexpensively improve the detection of diphtheritic neuropathy. Notably, diphtheritic neuropathy, which accounts for a minority of all diphtheritic illness, can signal the need for improved routine DTP or DTP combination vaccination coverage, lead to booster vaccinations for persons in various age groups, and herald the need for release of antitoxin from storage for administration to patients. Because antitoxin is not fully available and must be administered promptly, routine and bolstered immunization strategies remain crucial for reducing the incidence of diphtheria, and early detection through existing surveillance systems is especially needed for nonimmunized community populations.

## Study Strengths and Limitations

Although this study represents a convenience sample because of chart availability and may represent reporting bias, the case-patients reported had high case-fatality and disability rates at last known follow-up evaluation at 60 days. The cases have fairly complete information regarding onset and course of the neuropathy but lack details surrounding the first phase of the diphtheritic illness, infectious contacts, and community-level and person-level vaccination status. None of the case-patients reported, with the possible exception of 1 case-patient, represent definite diphtheria diagnosed without bacteriological identification. This finding is also common in outbreaks in middle-income countries because the organism may be difficult to identify (*19*,*21*–*24*). All cases in this report should be best considered as suggestive of diphtheria.

It is possible but unlikely that these case-patients had Guillain-Barré syndrome, poliomyelitis, or other causes of AFP (*25*,*26*). In addition to diphtheria, there are other differential diagnoses for AFP with respiratory involvement for patients in this age group. However, these case-patients displayed several classic features of diphtheria and meet the definition of having probable cases. Finally, AFP surveillance in India is targeted to persons <15 years of age. Diphtheria is not limited to persons in this age group and may be seen in adults, including older adults. In higher-income countries, eradication of diphtheria was first achieved among children in the youngest age groups and later older children and adults became more vulnerable because of waning immunity or incomplete immunization (*27*). A study specific to healthy adults in India found that adults in the oldest age group were least likely to have protective levels of antibodies against *C. diphtheria* (*28*).

Given resource constraints, the high reported mortality rate in this case series approximates the mortality rate for reports of diphtheria outbreaks in India in general but represents selection bias for more severe cases. The mortality rate was 73% in a case series from Assam and was maximal in persons 3–5 years of age (*27*). Another report for Assam found that 31% of patients with diphtheria died (*20*). These case series identified polyneuropathy in a minority of cases, suggesting that diphtheritic polyneuropathy in low-income settings might represent more severe presentations of the disease and, by the time of presentation, might be less responsive to standard interventions.

## Conclusions

The present study reports a sample of diphtheritic polyneuropathy that occurred in India over a 7-year time frame. Diphtheria represents a small proportion of AFP cases in India. AFP is the most severe presentation of polyneuropathy. If polyneuropathy also occurs in 20% of patients with diphtheria in this region, then surveillance of diphtheritic polyneuropathy by existing networks identifies a small but major fraction of severe diphtheria cases in India.

Diphtheria can be eliminated for several reasons: the reservoir is only humans; there is a safe, effective, and affordable vaccine; and seasonal outbreaks make transmission interruption possible (*27*). However, a low level of transmission of diphtheria, a fully vaccine-preventable disease, leaves the possibility for long-term reemergence of diphtheria in many countries in which vaccination has been inadequate. Asymptomatic carriers persist. Partial immunization; increased numbers of diphtheria cases in certain groups, such as women and certain religious groups; and changing demographic patterns, such as cases in older children, are all concerning features that lead to continued disease transmission (*13*). Poor awareness for additional protection of DTP and DTP combination vaccines compared with other vaccinations and societal and individual-level fatigue with vaccinations in general can reduce free and available vaccination against diphtheria (*19*). Late and missed diagnoses and limited expertise in sample collection may contribute to the high case-fatality rate. Case-based surveillance and mandatory booster vaccination at the time of primary school entry have been suggested for children in India (*13*).
